# Editorial: Brain cells' compensatory mechanisms in response to disease risk factors

**DOI:** 10.3389/fnmol.2022.1096287

**Published:** 2022-12-20

**Authors:** Yong Kim, Byung-Chang Suh

**Affiliations:** ^1^Department of Neurosurgery, Robert Wood Johnson Medical School, Rutgers University, Piscataway, NJ, United States; ^2^Brain Health Institute, Rutgers University, Piscataway, NJ, United States; ^3^Department of Brain Sciences, Daegu Gyeongbuk Institute of Science and Technology (DGIST), Daegu, South Korea

**Keywords:** brain cells, compensatory mechanism, homeostasis, risk factors, brain disorders

Our brain is highly plastic not only to sensory stimuli but also to environmental, chemical, and biological stressors. Molecules in brain cells must be altered and adapted in response to external challenges to maintain stability at the circuit and network levels and to behaviorally cope with external stressors or challenges. Similar adaptations are likely required in response to risk factors of brain disorders.

Brain plasticity or adaptation has been observed in response to stressful experiences (McEwen and Gianaros, 2011). Behavioral experience such as motor experience significantly affects the recovery of brain in either adaptive or maladaptive ways after brain injury (Nudo, 2013). Mechanical stress, i.e., traumatic brain injury causes multiple biochemical and cellular changes including intracellular trafficking, protein aggregation and complement activation (Surgucheva et al., 2014; Ng and Lee, 2019). In case of cancer therapy, intracellular adaptations of tumors or their adaptations to extracellular environment may lead to resistance against cancer drugs, resulting in transient or partial inhibition of tumor cell growth (Vaupel and Harrison, 2004; von Manstein et al., 2013). Maladaptation of brain reward system is implicated in drug addiction or persistent vulnerability to relapse (Koob and Le Moal, 2001; Ferland et al., 2019). Increased neuronal activity or hypermetabolism has been thought as a compensatory mechanism of neurodegeneration in Alzheimer's disease or Parkinson's disease (Ashraf et al., 2015; Blesa et al., 2017). In this regard, individual differences in molecular and cellular adaptations possibly drive susceptibility or resilience in response to stressors or risk factors of diseases as well as subsequent disease progression and/or vulnerability to relapse. Thus, studies of such compensatory mechanisms would provide a great opportunity of identifying disease mechanisms, new biomarkers and therapeutic targets.

Bhatti et al. used a chronic social defeat stress (CSDS) paradigm and searched critical cell types and molecular alterations involved in individual differences in stress responses in mice. They found parvalbumin (PV)-expressing GABAergic interneurons are altered in response to CSDS and their alterations are causally related to susceptibility or resilience to stress-induced social avoidance or anhedonia-like behavior. PV neuron-selective translational profiling indicates mitochondrial oxidative phosphorylation is the most significantly altered pathway in stress-susceptible versus resilient mice. Among differentially expressed genes associated with stress-susceptibility and resilience, the authors found alterations of *Ahnak* gene expression is causally related to stress-induced divergent behavioral adaptations. Notably, Ahnak was found as a major scaffolder of S100a10 and AnxA2 in the brain (Jin et al., 2020), and alterations of S100a10 is highly implicated in the pathophysiology of major depressive disorders and antidepressant actions (Svenningsson et al., 2013; Chen et al., 2022). Ahnak was also found as an endogenous regulator of L-type voltage-gated calcium channels (VGCCs) in the brain (Jin et al., 2020) and human genetic studies implicate altered function of L-type VGCCs in the pathophysiology of multiple psychiatric disorders including major depressive disorder, bipolar disorder, schizophrenia and autism spectrum disorder (Green et al., 2010; Liu et al., 2011; Bhat et al., 2012; Cross-Disorder Group of the Psychiatric Genomics [Corporate Author], 2013; Schizophrenia Working Group of the Psychiatric Genomics Consortium, 2014; Pinggera et al., 2015). Thus, their findings might be relevant to the pathophysiology of neuropsychiatric disorders.

Autism spectrum disorder (ASD), as a neurodevelopmental and neuropsychiatric disorder, is characterized by impaired social communication, restricted interests and elevated repetitive behaviors (Lord et al., 2018, 2020). Because ASD is affected by multigenic traits, genetic polymorphism in multiple genes in affected individuals may influence resilience or susceptibility to ASD (Bourgeron, 2015). Lim, Yoon et al. reviewed ASD-related genes and their distinctive signaling pathways and dysfunction relevant to a variety of autism spectrum-related phenotypes. In addition, systematic review on existing animal models of ASD is also provided. ASD has been linked to genes involved in synaptic transmission and scaffolding, chromatin remodeling, protein synthesis and degradation, and actin cytoskeletal dynamics, all of which are highly important for neuronal adaptations or synaptic strength or scaling (Bourgeron, 2015; Lee et al., 2017; Tatavarty et al., 2020). Thus, this review article provides insight into potential roles of adaptive mechanisms or synaptic plasticity in this multifactorial brain disorder.

In a separate research article, Lim, Kim et al. investigated potential interaction between lysophosphatidic acid (LPA) receptor-mediated pathway and dendritic deficits in a cell model of ASD. They have found that gintonin, a substance isolated from ginseng, has an effect on the dendritic growth of cultured striatal neurons. Gintonin is a lipoprotein composed of LPA and ginseng protein, and its effect is mediated *via* the LPA receptor. In their study, the loss-of-function of Slitrk5 or Shank3 genes-mediated reduction in dendritic complexity in primary striatal neurons was restored by gintonin treatment *in vitro*. Although further studies with an *in vivo* model should be complemented, this study implicates ASD-relevant deficits in neuronal development might be reversible or plastic in response to extracellular signaling molecules such as LPA.

Small, non-coding RNAs called microRNAs (miRNAs) inhibit the function of protein-coding transcripts, and thereby regulates various aspects of brain function including synaptic development and transmission as well as neuronal survival (Cho et al., 2019; Brennan et al., 2020). Bai et al. investigated the roles of miR-29a/b1 in aging and Parkinson's disease (PD). While miR-29a/b1 knockout mice display accelerated aging in the periphery, deletion of miR-29a/b1 alleviates MPTP-induced neuronal damages, glial activation and behavioral impairments. Interestingly, they observed an increase of miR-29a levels in the cerebrospinal fluid of PD patients compared to the levels in healthy subjects as well as in cultured microglia, glia and neurons treated with LPS or MPP+, a neurotoxin. It is intriguing to imagine that miR-29a might be initially elevated as a part of cellular compensatory mechanisms, but eventually aggravating disease progression. Further exploration of downstream targets and understanding the function of elevated miR-29a in specific cell types are warranted.

In summary, the four articles contributed by Bhatti et al., Lim, Yoon et al., Bai et al., Lim, Kim et al. in this Research Topic exemplify a great potential of studies of brain cells' compensatory mechanisms for identifying disease mechanisms, therapeutic targets or biomarkers. Because this Research Topic can be broadly applicable to a variety of biological systems, many new research avenues can be explored under the scope of this Research Topic in the future.

## Author contributions

Both authors listed have made a substantial, direct, and intellectual contribution to the work and approved it for publication.
